# Pathogens in ticks collected from dogs in Berlin/Brandenburg, Germany

**DOI:** 10.1186/s13071-014-0535-1

**Published:** 2014-12-02

**Authors:** Cécile Schreiber, Jürgen Krücken, Stephanie Beck, Denny Maaz, Stefan Pachnicke, Klemens Krieger, Marcus Gross, Barbara Kohn, Georg von Samson-Himmelstjerna

**Affiliations:** Institute for Parasitology and Tropical Veterinary Medicine, Freie Universität Berlin, Berlin, Germany; Small Animal Clinic, Freie Universität Berlin, Berlin, Germany; Bayer Vital GmbH, Leverkusen, Germany; Bayer Animal Health GmbH, Monheim, Germany; Institute for Statistics and Economy, Freie Universität Berlin, Berlin, Germany; Institute of Immunology, Freie Universität Berlin, Berlin, Germany

**Keywords:** Canine vector-borne diseases, *Borrelia*, *Babesia*, *Rickettsia*, *Anaplasma*, *Candidatus* neoehrlichia mikurensis

## Abstract

**Background:**

Tick-borne diseases are a major health risk for humans and dogs. In addition to collection and analysis of questing ticks, analysis of host-associated ticks for the presence of pathogens is a valuable method to gain insight into transmission patterns of tick-borne diseases.

**Methods:**

Ticks were collected from dogs living in the Berlin/Brandenburg area. The three tick species *Ixodes ricinus, Ixodes hexagonus* and *Dermacentor reticulatus* were examined for the presence of *Babesia* spp., *Borrelia* spp., *Rickettsia* spp. and Anaplasmataceae. Conventional PCR followed by sequencing was used for pathogen detection and characterization.

**Results:**

*Babesia* spp. were found in 2.5% and 3% of *I. ricinus* and *I. hexagonus*, respectively. Sequencing revealed the presence of *Babesia microti, Babesia capreoli* and *Babesia venatorum. D. reticulatus* were free of *Babesia canis. Rickettsia* spp. were detected in 61% of *I. ricinus*, 44% of *I. hexagonus* and 39% of *D. reticulatus.* Specifically detected were *Rickettsia raoulti* in *D. reticulatus* and *I. hexagonus, Rickettsia helvetica* in *I. ricinus* and *I. hexagonus* and *Rickettsia monacensis* in *I. hexagonus. Anaplasma phagocytophilum* and *Candidatus* Neoehrlichia mikurensis have been reported previously in *I. ricinus* (6.5% and 4.3%, respectively) and *I. hexagonus* (3.9% and 5.9%). *Borrelia* spp. were found in 11.6% of *I. ricinus* and 11.2% of *I. hexagonus.* Subsequent genospecies analysis revealed *Borrelia afzelii, Borrelia garinii, Borrelia burgdorferi* sensu stricto and *Borrelia miyamotoi.* Simultanous presence of more than one pathogen was found in 20% of *I. ricinus* and in 59% of *I. hexagonus* whereas the total frequency of any pathogen was 65% in *I. ricinus,* 59% in *I. hexagonus* and 64% in *D. reticulatus.* Ticks in which *A. phagocytophilum* was detected had a significantly increased risk of also containing *Rickettsia*. Ticks harbouring a pathogen had significantly higher scutal indices than ticks without presence of any pathogen.

**Conclusions:**

Frequencies of potential human or canine pathogens in ticks were considerable and DNA of all four groups of pathogens was detected. Differences in scutal indices might suggest that pathogens are frequently taken up by ticks when feeding on dogs in Berlin/Brandenburg.

**Electronic supplementary material:**

The online version of this article (doi:10.1186/s13071-014-0535-1) contains supplementary material, which is available to authorized users.

## Background

Vector-borne diseases represent an important threat to canine health and are also of major zoonotic relevance [1]. Many tick species are potential vectors of infectious agents that are pathogenic in dogs. In the area of Berlin/Brandenburg, the most common tick species reported to date are *Ixodes ricinus, Ixodes hexagonus* and *Dermacentor reticulatus* [2]. Important endemic tick transmitted pathogens in Germany are species of *Babesia*, *Borrelia*, *Rickettsia* and members of the Anaplasmataceae.

Intraerythrocytic parasites of the genus *Babesia* are frequently found in mammalian hosts, although the individual parasite species usually have a quite restricted host spectrum [3]. Studies in Germany have reported a prevalence of *Babesia* of 1–4.1% in *I. ricinus*, predominantly the zoonotic *Babesia microti, Babesia venatorum* and *Babesia divergens*, but also *Babesia capreoli* [4,5]. In southern Germany, 2.5% of *D. reticulatus* ticks contained the non-zoonotic canine parasite *Babesia canis* [6].

*Borrelia* spp. belong to the gram-negative bacteria of the order Spirochaetales. The most common agents are Lyme disease pathogens belonging to the *Borrelia burgdorferi* sensu lato complex, a group of at least 16 different genospecies, such as *Borrelia burgdorferi* sensu stricto, *Borrelia spielmanii, Borrelia afzelii, Borrelia garinii, Borrelia valaisiana, Borrelia lusitaniae* and *Borrelia bavariensis* [7,8]*.* The relapsing-fever *Borrelia*, such as *Borrelia miyamotoi, Borrelia hispanica* and *Borrelia persica* are known to be endemic in Europe as well [9]. *Borrelia miyamotoi* was found in ticks from Germany [10] and in ticks from France [11].

*Rickettsia* spp. are obligate intracellular α-proteobacteria belonging to the order Rickettsiales [12]. In German ticks, prevalences of 14.2% have been reported for *R. helvetica* in *I. ricinus* and 30% for *R. raoulti* in *D. reticulatus* [13,14]. In another study from Germany, a prevalence of 8.6% for *R. monacensis* was found in *I. ricinus* [15].

As another family in the order Rickettsiales, members of the Anaplasmataceae are frequently found throughout Europe. Important members of the Anaplasmataceae are *Anaplasma phagocytophilum* and *Candidatus* Neoehrlichia mikurensis in central and northern areas and *Ehrlichia canis* and *Anaplasma platys* in mediterranean areas [10,16]. *Candidatus* Neoehrlichia mikurensis is considered to be an emerging pathogen, first identified in *I. ricinus* in the Netherlands and described as *Ehrlichia-*like organism [17]. Subsequently, this pathogen was found in wild rats and *Ixodes ovatus* ticks in Japan [18] and given its name *Candidatus* Neoehrlichia mikurensis. The pathogenicity of this organism for humans was revealed in febrile patients living in Germany [19], Sweden [20] and Switzerland [21]. Dogs also appear to be affected by infections [22]. To date *Candidatus* Neoehrlichia mikurensis was found in European *I. ricinus* and *I. hexagonus* ticks [23]. Besides *I. ovatus* from Japan [18] also *Rhipicephalus* spp. and *Haemaphysalis* spp. from Nigeria [24] and *Ixodes persulcatus* from Russia [25] were shown to contain DNA of this pathogen. Prevalences of 8.1% and 10.7% were reported in Germany and the Czech Republic, respectively, whereas *Candidatus* Neoehrlichia mikurensis was found in only 1.7% of French ticks [10].

All above mentioned pathogen groups are known to be potentially pathogenic for dogs and humans. The successive findings of emerging infectious diseases substantiate the importance of studies of ticks. The aim of the present study was to determine the frequency of pathogens in dog-associated ticks to evaluate the current risk of infection for dogs living in Berlin and Brandenburg area.

## Methods

### Sample collection

In total, 1728 ticks (99.6% adults) were collected from 441 dogs at the Small Animal Clinic, Freie Universität Berlin, Germany as described in an earlier publication [26]. One of the ticks was accidentally lost. Participating owners collected ticks from their dogs during a time span of up to 13 months (1^st^ of March 2010 to 31^st^ of March 2011) and stored them in tubes containing 80% (v/v) ethanol. The ticks had been categorised in terms of species, stage and sex in the earlier study [26].

### DNA extraction

Tick DNA was extracted using two methods. Initially, the NucleoSpin® 96 Blood Kit (Macherey & Nagel) was used according to instructions provided by the manufacturer and DNA was collected in 50 μl elution buffer. Subsequently, the majority of ticks were extracted with the Maxwell® 16 LEV Blood Kit in the Maxwell® 16 instrument (Promega). Ticks were crushed in 400 μl lysis buffer and 30 μl Proteinase K were added. Tubes were then incubated at 65°C for 10 min before another 200 μl lysis buffer were added. Samples were then placed in the Maxwell® 16 instrument and the protocol for isolation of DNA from blood was started. DNA was collected in 50 μl elution buffer. After isolation, DNA concentration was measured by determining the optical density of samples using a Take 3 plate in a Synergy 4 plate reader (Biotek). DNA samples were stored at −80°C until further use. The standard DNA amount used for PCR was 50–140 ng in 1 μl. Samples with higher DNA concentration were diluted, for samples with a lower DNA concentration up to 5 μl were used as template. The minimum amount of DNA used in PCR was 50 ng in 5 μl.

### Determination of scutal index

All ticks were subjected to scutal index measurements, except 33 ticks that were not intact anymore. Pictures of every tick were taken with a Leica DFC360FX camera [26]. On those pictures body length, starting at the basis capituli, and scutum width at the scutums widest point were measured. The scutal index as the quotient of body length and scutum width was calculated [27].

### PCR assays and sequencing

The target region for the *Babesia* PCR was the 18S rRNA gene [28] (Additional file 1: Table S1). DNAs of 497 ticks collected from 131 dogs were subjected to PCR. Reactions contained 2.5 mM MgCl_2_, 0.2 mM dNTPs, 0.3 μM of each primer, 0.04 U/μl Maxima® Hot Start *Taq* DNA Polymerase (Thermo Scientific) and template DNA in 25 μl 1× Hot Start PCR buffer. Initial denaturation at 94°C for 4 min was followed by 40 cycles of 94°C for 15 s, 63°C for 30 s and 72°C for 40 s. Finally, samples were incubated at 72°C for 10 min. As positive controls, PCRs containing 25 copies of plasmid with the *Babesia gibsoni* target region as well as no-template controls were routinely run in parallel. Negative controls without template DNA were conducted in all PCR runs. The limit of detection as determined during setting up the PCR was ≤10 copies*.* All positive samples were purified from agarose gels and sent in for sequencing by GATC Biotech.

The PCR for detection of *Borrelia* spp. in 938 ticks collected from 211 dogs targeted the *hbb* gene [29] (for primer sequences see Additional file 1: Table S1). In this PCR, a 153 bp DNA fragment was amplified, with a detection limit of ≤5 copies. PCRs contained 0.2 mM dNTPs, 0.5 μM of each primer, 0.2 μl Phusion® Hot Start II High-Fidelity DNA Polymerase (Thermo Scientific) and template DNA in 20 μl 1× Phusion® HF Buffer. After initial denaturation at 98°C for 30 s, 40 cycles of 98°C for 5 s, 57°C for 30 s and 72°C for 10 s were perfomed, followed by a terminal elongation at 72°C for 5 min. As positive controls, PCRs containing 50 copies of plasmid with the *B. afzelii* target region were run in parallel. Of all positive samples, 67 *I. ricinus* samples and 13 *I. hexagonus* were sent in for sequencing.

In addition, a nested PCR targeting the 16S rRNA gene was used for two samples which could not be unequivocally identified using the *hbb* gene [11]. Reactions contained 0.16 mM dNTPs, 0.3 μM of each primer, 0.25 μl Phusion® Hot Start II High-Fidelity DNA Polymerase and template DNA in 25 μl 1× Phusion® HF Buffer. After initial denaturation at 94°C for 1 min, 50 cycles of 94°C for 20 s, 63°C for 20 s and 72°C for 40 s were followed by a final extension at 72°C for 2 min. For nested PCR, 1 μl of the PCR product of the first PCR was used as template and the annealing temperature was set to 56°C.

Two different PCRs were used for detection of *Rickettsia* spp. (for primer sequences see Additional file 1: Table S1) targeting 203 bp (to determine the frequency of *Rickettsia* in the ticks) and 676 bp (to identify the genospecies by sequencing) of the *gltA* gene. The first PCR (203 bp amplicon) was conducted on DNAs of 628 ticks collected from 147 dogs*.* This PCR contained 0.2 mM dNTPs, 0.5 μM of each primer, 0.25 μl Phusion® Hot Start II High-Fidelity DNA Polymerase (Thermo Scientific) and template DNA in 25 μl 1× Phusion® HF Buffer. After initial denaturation at 98°C for 30 s, 50 cycles with 98°C for 30 s, 52°C for 30 s and 72°C for 15 s were performed, followed by a final elongation at 72°C for 5 min. The detection limit was ≤5 copies. Positive samples were re-analysed in a second PCR containing 1.5 mM MgCl_2_, 0.5 μM of each primer (Rmasglta1065lo and CS409d), 0.2 mM dNTPs, 0.04 U/μl Maxima® Hot Start *Taq* DNA Polymerase and template DNA in 25 μl 1× Hot Start PCR Buffer. The detection limit was ≤10 copies. The template in positive controls contained 40 and 50 copies of a plasmid with the *R. raoulti* target sequence (for analysis of *Ixodes* samples) or the *R. helvetica* sequence (for *D. reticulatus* samples) in the first and second *Rickettsia* PCRs, respectively. Sixteen postive samples of *I. ricinus,* 19 positive samples of *I. hexagonus* and 12 positive samples of *D. reticulatus* were sent in for sequencing.

### Statistical analysis

Statistical analysis was performed using the R software and the R package “Epidemiological tools” [30]. Coinfection rates between two pathogens were calculated via Mid-P Exact, Chi square or Fisher-Exact test. Correlation between the infections of ticks with three pathogens was analysed via Chi square test and Fisher-Exact test. The correlation between tick species and probability of an infection was tested by Mid-P Exact test. Results of scutal index measurements as a tool to estimate the duration of the bloodmeal from each tick were published in a previous study [26] and were compared here between non-infected and infected female ticks using the Student’s t test and illustrated in a graph.

### Phylogenetic analysis

For the phylogenetic analysis of *Borrelia* spp., *hbb* nucleotide sequences were initially aligned using Clustal X2 default parameters [31]. The optimum nucleotide substitution model was identified with jModelTest 0.1 [32] with eight substitution rate categories. The phylogenetic tree was calculated by PhyML 3.01 [33] assuming the TPM3uf model. Substitution rates used were calculated in jModelTest and set to A- > C = C- > G = 42.62375, A- > G = C- > T = 409.92731 and A- > T = G- > T = 409.92731. The Γ shape parameter for distribution of the eight substitution rate categories, the number of invariable positions and the nucleotide frequencies were calculated and optimised using PhyML. The calculation of the optimium tree was started with five random trees and one neighbour joining tree. The statistical support for the branches was obtained from the Shimodaira-Hasegawa modification and a Bayesian transformation of the approximate likelihood ratio test.

## Results

### Frequencies of individual pathogens in dog associated ticks

The data for individual ticks including the hosts from which they were collected are provided in Additional file 2: Table S2. Moreover, (Additional file 3: Table S3) summarises the data of those ticks that were analysed for the presence of all pathogens.

The frequency of *Babesia* spp. was 2.5% in *I. ricinus,* 3% in *I. hexagonus* and 0% in *D. reticulatus* (Table 1)*.* The five PCR-positive samples of *I. ricinus* were further analysed and three *B. microti,* one *B. venatorum* and one *B. capreoli* were identified. Sequencing results for *I. hexagonus* samples revealed exclusively *B. venatorum* (n = 3)*.*

**Table 1:** **Detection frequencies of analysed tick species**

**Tick species**	***Babesia*** **spp. (95% CI** ^**a**^ **)**	***A. phago-cytophilum*** **(95% CI)**	***Candidatus*** **Neo-ehrlichia mikurensis (95% CI)**	***Rickettsia*** **spp. (95% CI)**	***Borrelia*** **spp. (95% CI)**
***I. ricinus***	2.5 (0.92-5.54) n = 200	6.5 (4.9-8.4) n = 774	4.3 (3.0-5.9) n = 774	61.0 (54.2-67.5) n = 205	11.6 (9.5-14.0) n = 768
***I. hexagonus***	3 (0.77-7.95) n = 100	3.9 (1.6-8.0) n = 152	5.9 (2.9-10.6) n = 152	44.4 (36.6-52.4) n = 151	11.2 (6.9-17.0) n = 152
***D. reticulatus***	0 (0.00-1.51) n = 197	n. d.^b^	n. d.	39.3 (33.7-45.2) n = 272	n. d.

^a^95% confidence interval.

^b^not determined.

For *Borrelia* spp., PCR detected DNA of these pathogens in 11.6% for *I. ricinus* and 11.2% for *I. hexagonus* (Table 1). Sequencing of the *hbb* amplicon was performed for 67 *I. ricinus* and 13 *I. hexagonus* samples*.* Thirteen *I. ricinus Borrelia* spp. PCR-positive samples were assigned to *B. afzelii,* 19 to *B. garinii* and 24 to *B. burgdorferi* s.s. *Borrelia* spp. positive samples of *I. hexagonus* were identified as two *B. garinii*, three *B. afzelii* and five *B. burgdorferi* s.s. Eleven samples of *I. ricinus* and three samples of *I. hexagonus* had the closest relationship to *Borrelia turicatae* based on the *hbb* sequence (88% identity with 100% coverage). Since identity to *B. turicatae* was much lower than 100%, *B. turicatae* has not been described to be present in Europe and no sequences of the closely related *B. miyamotoi* were available for the *hbb* gene, another PCR was conducted for two of the 14 identical *B. turicatae*-like samples. Using a nested PCR detecting the 16S rDNA of *Borrelia* spp. followed by sequencing and BLASTn analysis, *B. miyamotoi* was identified in these samples. All 14 samples were thus considered to be *B. miyamotoi*. The sequence of *B. miyamotoi* for the *hbb* gene was deposited in the database of the European Bioinformatics Institute (www.ebi.ac.uk) with the accession number HE993870. The result of the phylogenetic analysis is shown in Additional file 4: Figure S1.

The DNA of *Rickettsia* spp. was found in 61% for *I. ricinus,* 44.4% for *I. hexagonus* and 39.3% for *D. reticulatus* (Table 1)*.* Sequencing of 16 PCR-positive samples of *I. ricinus* confirmed *R. helvetica* in all samples*.* In 19 positive samples of *I. hexagonus*, one *R. raoulti,* two *R. monacensis* and 16 *R. helvetica* were detected. All 12 DNA samples of *D. reticulatus* that were subjected to sequencing revealed *R. raoulti.*

### Analysis of simultaneous presence of multiple pathogens in ticks and overall frequency of pathogens

For analysis of simultaneous presence of multiple pathogens in single ticks, also previously published results analysing all *Ixodes* spp. ticks collected in this study (926 ticks from 210 dogs of the study) for Anaplasmataceae were included; that is PCR detected Anaplasmataceae in 10.7% of *I. ricinus* and 9.9% of *I. hexagonus* [23]. Herein, pathogen detection was performed either by conventional PCR followed by Sanger sequencing or by PCR with high-resolution melting curve analysis. Of all examined *I. ricinus* (n = 774) and *I. hexagonus* (n = 152) samples 6.5% and 3.9% were positive for *A. phagocytophilum,* respectively. Frequencies for *Candidatus* Neoehrlichia mikurensis were 4.3% in *I. ricinus* and 5.9% in *I. hexagonus.*

**Figure 1:**
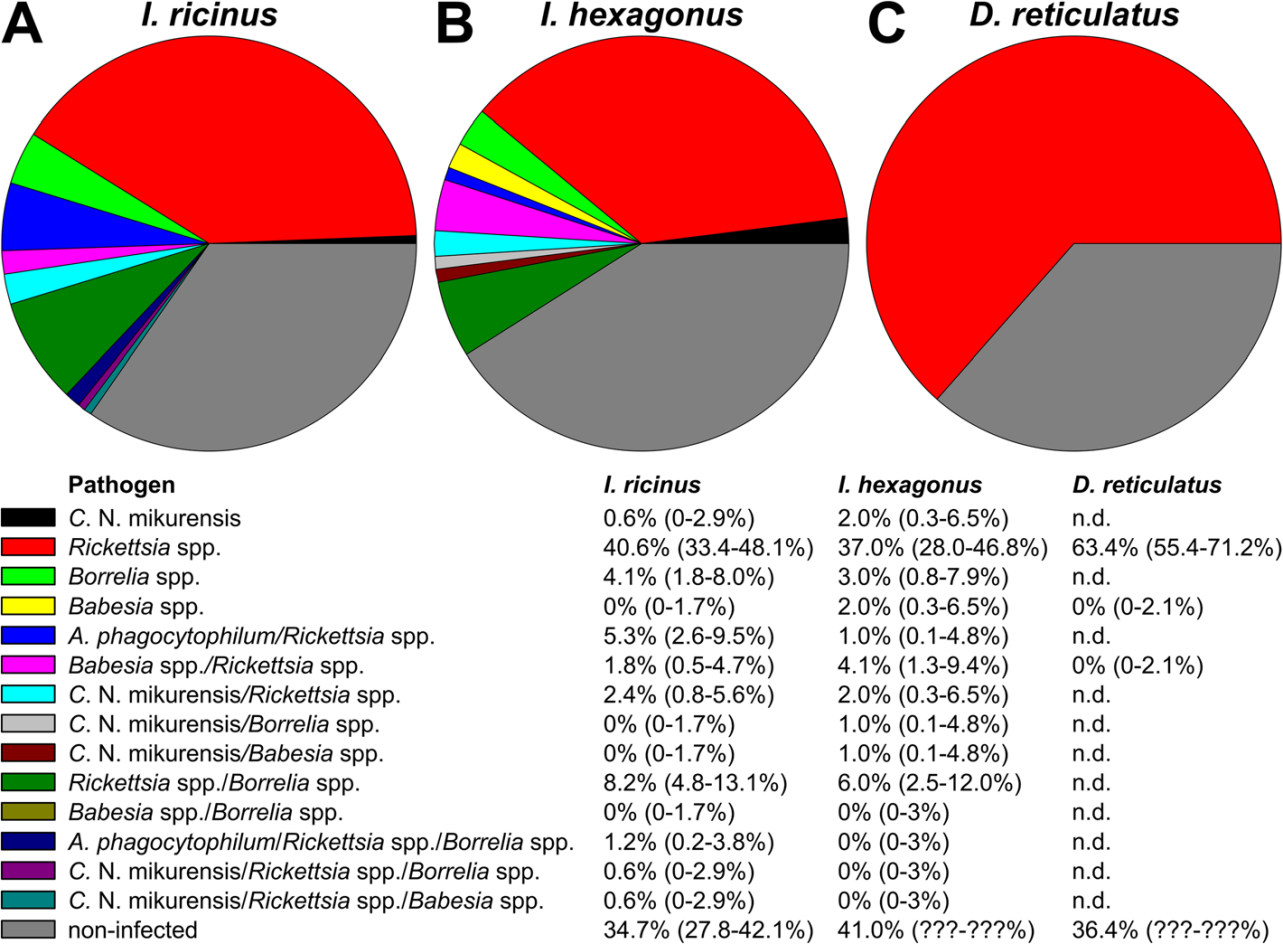
**Frequencies of pathogen detection in** ***Ixodes ricinus*** **(n = 170) (A),** ***Ixodes hexagonus*** **(n = 100) (B) and** ***Dermacentor reticulatus*** **(n = 140) (C) examined for all pathogens.** n. d., not determined. Below the pie charts mean frequencies (95% confidence intervals) are given.

Simultanous presence of two or more pathogens was detected in 20.2% of *I. ricinus* (CI: 14.5-26.5%, n = 170, Figure 1). Overall, at least one pathogen was detected in 65.0% (CI: 57.9-72.2%) of the 170 *I. ricinus* examined for presence of all pathogens. More than one pathogen was detected in 15.0% (CI: 9.0-23.0%) of 100 *I. hexagonus* that were examined for all pathogens (Figure 1). The overall presence of pathogens for these ticks was 59.0% (CI: 49.2-68.3%). In 140 *D. reticulatus* examined for *Rickettsia* spp. and *Babesia* spp. both pathogens were detected in 0% (CI: 0.0-2,1%, Figure 1), since the only pathogens that were found in this tick species were *Rickettsia* spp. Therefore, the overall frequency of pathogens was identical to the presence of *Rickettsia* spp. in *D. reticulatus* being as high as 63.6% (CI: 55.4-71.2).

There was a significant difference between tick species in their likelihood to be positive for pathogens. *Ixodes hexagonus* was more likely to contain pathogens than *D. reticulatus* (p = 1.8 × 10^−11^; odds ratio 0.13) or *I. ricinus* (p < 0.002; odds ratio 0.39).

The examination of correlations between the presence of two pathogens in *I. ricinus* and *I. hexagonus* was conducted using the Mid-P Exact test. There was a significant correlation between the infections with *A. phagocytophilum* and *Rickettsia* spp. (p < 0.05; odds ratio >7.771) in *I. ricinus*. All other combinations of pathogens were tested for both tick species but showed no significant correlations.

### Effects of the feeding-time on the probability to detect pathogens

**Figure 2:**
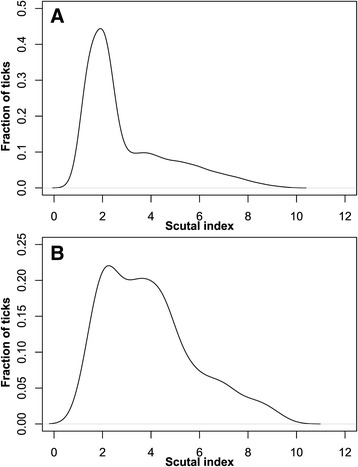
**Density curve of scutal index (SI) for female non infected (A) and pathogen-positive (B) ticks.** X-axis shows SI values and y-axis fraction of ticks.

Infected ticks had a higher mean SI than non-infected ticks (data from all three species together). The median SI of non-infected ticks was 2.2, the mean was at 3.0. The median and mean SIs for infected ticks were 3.6 and 3.9, respectively. This difference was statistically significant according to t-test results (p = 2.2 × 10^−7^). For the individual tick species the difference in SI means between ticks being tested positive and ticks being tested negative for pathogens was only significant for *D. reticulatus* (mean SI 2.7 vs. 3.7, p < 0.0005). Two separate graphs, showing density plots for ticks tested negative for pathogens (Figure 2A) and ticks tested positive for pathogens (Figure 2B), were drawn to illustrate the percentage of ticks (y-axis) featuring a certain SI (x-axis). The curve for ticks containing no pathogens (Figure 2A) has a maximum at an SI of approximately 2, which corresponds to a blood meal duration of approximately one day [34]. The curve for ticks containing at least one pathogen (Figure 2B) has a plateau at an SI range of 2–5 corresponding to blood meal durations of 1–5 days [34].

### Potential aggregation effects

Since the number of collected ticks was of course much higher than the number of dogs in the study and due to the increased probability to detect pathogens in ticks with high SI, it is possible that data were biased by many ticks containing pathogen DNA that originated from the same infected dog. Therefore, results regarding ticks from those four dogs, for which the highest numbers of ticks were sent in, are reported in the following. However, no statistical analysis was conducted since the number of ticks from these four dogs was too small compared to the number of ticks from the remaining dogs. Furthermore, the latter also contained data of ticks collected from individual dogs. From the first dog, 24 ticks (all *Ixodes* sp.) were collected and pathogens were detected in 15 (62.5%) of these, including 10 *Rickettsia* spp. (41.7%), three with Anaplasmataceae (12.5%), and five with *Borrelia* spp. (20.8%). The latter represents a remarkably high infection rate compared with the approximately 11% frequency of *Borrelia* spp. found in all *Ixodes* ticks. Of the 35 ticks sent in from the second dog, 17 were *Ixodes* spp. and 18 were *D. reticulatus*. Six out of 15 analysed *D. reticulatus* (40%) were positive for *Rickettsia* spp. *Rickettsia* spp. were also detected in 7 of 13 *Ixodes* spp. (54%) while in three out of 16 analysed *Ixodes* spp. (19%) Anaplasmataceae were found which is approximately twice as high as in the overall *Ixodes* ticks in the present study. From a third dog, 22 *Ixodes* spp. ticks were sent in and 10 of these (45%) were positive for *Borrelia* spp. which is again unusually high. In addition, in 6 out of 11 tested ticks *Rickettsia* DNA was detected (54%). Finally, from a fourth dog 27 *Ixodes* and 28 *Dermacentor* were obtained. Within the *Ixodes* group, 8 of 27 (29.6%) and 2 of 25 (8%) were positive for Anaplasmataceae and *Borrelia* sp., respectively. Among 16 tested *D. reticulatus*, *Rickettsia* spp. DNA was detected in 2 (12.5%). In this case, the number of Anaplasmataceae-positive *Ixodes* ticks was unexpectedly high.

## Discussion

The salient feature of this study is the examination of host-associated ticks. Most previous studies used questing ticks obtained by flagging, a technique that can sample only small areas and is not suitable to obtain data on a larger geographic scale. Dogs have recently been proposed as sentinels for human tick exposure, however this study did not sample ticks from dogs or analyse ticks for the presence of pathogens [35]. The ticks analysed in the present study were sampled directly from dogs in the Berlin/Brandenburg area and were a mixture of unattached and partially or nearly fully engorched specimen [26]. Although they did not cover the Berlin city area in a representative manner, they definitely exhibit a broader distribution than those obtainable by flagging of individual small habitats.

Dogs were used as sentinels to estimate the risk of human Lyme borreliosis in serological surveys in the USA [36,37] and the UK [38]. In a very special setting with *Ixodes scapularis* carrying *B. burgdorferi* s.l. invading an area, which was previously free of this vector, Hamer et al. [39] compared serological assays using canine serum with PCR assays to detect presence of pathogens in dog-associated ticks and found that analysis of ticks was superior. In general, however, it cannot be expected that frequency of pathogens in partially or fully engorged ticks will be the same as in questing ticks in the same area. Indeed, Leschnik et al. [40] found different tick communities and pathogen patterns when comparing dog-associated and questing ticks from the same area. Reasons for such differences can be uptake of parasites during feeding from an infected host or by co-feeding with an infected tick as well as aggregation of ticks on a few individual hosts that differ significantly in their infection status from the overall dog population in the study. Moreover, it must be kept in mind that detection of a pathogen in a feeding tick does not necessarily mean that the tick is or becomes truly infected since pathogens taken up via the bloodmeal but not able to replicate in the particular tick species will also be detected. The number of studies analysing host associated ticks for the presence of pathogens is rather limited today and ticks in these studies were either collected from deer [34], from a wide range of different domestic animals [41] or from dogs [39,40,42].

Several previously published studies in Europe have reported prevalence rates for the tick transmitted pathogens investigated here. Since no dog-specific *Babesia* spp. were detected in the present study and infection by co-feeding is probably not possible for *Babesia*, since ticks cannot be infected with sporozoites, the data for *Babesia* are probably relatively representative for those in questing ticks. Pathogen frequencies of 2.5% and 3% for *Babesia* spp. in *I. ricinus* and *I. hexagonus* in Berlin were similar to prevalences reported in other studies in Europe analysing questing ticks. In Europe, the prevalence of these pathogens ranged between 0.9% (Norway) and 7.4% (Slowenia) [4,43–46]. In Berlin, the *Babesia* species detected by sequencing were *B. microti* (1.5%) and *B. venatorum* (0.5%) as well as *B. capreoli* (0.5%) in *I. ricinus*, whereas in *I. hexagonus* all positive samples revealed to be *B. venatorum* (3%). These findings are in accordance with those reported from questing ticks in Leipzig, where the predominant species were identical. In a study analysing *I. ricinus* collected from domestic animals in the Netherlands, frequencies of 1.2% for *B. venatorum* and of 0.4% for *B. divergens* and *B. microti* were determined [41]. In this study all five *Babesia* positive ticks were obtained from dogs*.* In the same study, no *Babesia* spp. were detected in *I. hexagonus* [41]*.* The vector competence of this tick species could not be confirmed [47]. However, vector competence appears to be dependent on the *Babesia* strain [48]. Since *Babesia* spp. were found in *I. hexagonus*, it can currently not be excluded that *I. hexagonus* is a vector for *Babesia* spp. *Babesia microti* as well as *B. venatorum* and the closely related *B. divergens* (not found in Berlin in the present study) are known to be zoonotic pathogens [49,50] but they most likely do not impose any health risk to dogs. However, very closely related pathogens, called *B. microti*-like or *Theileria annae*, have been reported in sick dogs [51]. *Babesia capreoli* is not considered to be a health risk pathogen for either humans or dogs. Since no *B. canis* were detected in *D. reticulatus* this important canine pathogen has apparently not been introduced in the tick population in Berlin/Brandenburg yet.

Similar to *Babesia* spp., the frequency of *Borrelia* spp. in the dog-associated ticks in the present study is in the range found within previous European studies. Here, *Borrelia* spp. were found in 11.2% and 11.6% of *I. hexagonus* and *I. ricinus*, respectively. The prevalences in questing ticks in other European studies differ dramatically ranging from 7.6% to 40% [10,52,53]. In ticks collected from domestic animals in the Netherlands, *B. burgdorferi* s.l. were detected in 7.2% of all samples, which were predominantly derived from dogs. In the samples from Berlin analysed here, *B. afzelii, B. garinii* and *B. burgdorferi* s.s. were detected, all of them belonging to the Lyme Borreliosis group which are pathogens for both, dogs and humans. *Borrelia miyamotoi* is not known to cause disease in dogs but is pathogenic to humans [54]. Knowledge regarding the occurrence of this pathogen is rather new for Germany as it was only found two times in *I. ricinus* [10,11]. To the authors knowledge this is the first time that *B. miyamotoi* has been detected in *I. hexagonus.*

In contrast to the frequency of *Babesia* spp. and *Borrelia* spp., the frequency of *Rickettsia* spp. in ticks collected from dogs in this study was higher than in most previous reports for this pathogen group. In 39% of *D. reticulatus*, *Rickettsia* spp. were detected, whereas *I. ricinus* and *I. hexagonus* were positive in 61% and 44%, respectively. Previously published reports of *Rickettsia* spp. in questing *D. reticulatus* in Germany described prevalences as high as 23% or 30% in ticks collected from deer [13,34]. The published prevalence of *Rickettsia* spp. in *I. ricinus* from Germany was 14.2% [14]. *Ixodes hexagonus* from the Netherlands collected from companion animals were infected at a rate of only 0.8% [41]. In the present study, *R. helvetica* and *R. raoulti* were the only genospecies detected in *I. ricinus* and *D. reticulatus*, respectively. In contrast, *I. hexagonus* were positive for *R. helvetica*, *R. monacensis* and *R. raoulti*. This distribution confirms previously published studies reporting that *R. raoulti and R. helvetica* are the main species found in deer-associated *D. reticulatus* and questing *I. ricinus*, respectively [15,34]. *Rickettsia monacensis* was also previously found in *I. ricinus* [55,56]. The unexpectedly high frequency of *Rickettsia* spp. in ticks from Berlin is particularly important, because of their potential pathogenicity in humans, as described in previous studies [11,57–59]. To date no clinical illness in dogs has been related to those *Rickettsia* species detected in Germany, but systematic approaches to address pathogenicity of these infectious agents in animals are lacking.

Anaplasmataceae, as pathogens for dogs and humans, were detected with a rate of 10.7% in *I. ricinus* and 9.9% in *I. hexagonus* from this study [23]. They were assigned to two species, namely *A. phagocytophilum* and *Candidatus* Neoehrlichia mikurensis. *A. phagocytophilum* was found at a prevalence of 6.5% in *I. ricinus* and 3.9% in *I. hexagonus.* These frequencies of *A. phagocytophilum* matches with other previously published reports from questing *Ixodes ricinus* in Germany with 2.2 and 6.5% [41,60,61]. In the Netherlands, *A. phagocytophilum* was detected in 5.9% of *I. hexagonus* (all positive ticks collected from three hedgehogs and in 1.6% of *I. ricinus* (positive ticks collected from one cat and three dogs) [41]. The same applies to the prevalence for *Candidatus* Neoehrlichia mikurensis in Berlin, which was found in 4.3% and 5.9% of *I. ricinus* and *I. hexagonus*, respectively. Recently, this emerging pathogen has been found in 8.1% of German *I. ricinus* [10] and it may cause diseases in humans [19] and in dogs [22]. In the study from the Netherlands *Candidatus* Neoehrlichia mikurensis was found in 2.4% of all animal-associated *I. ricinus* (six ticks collected from one cat and five dogs).

*Dermacentor reticulatus* specimen were not tested for Anaplasmataceae and *Borrelia* spp. since a parallel study (M. Kohn, J. Demeler, J. Krücken, G. von Samson-Himmelstjerna et al., unpublished data) revealed a prevalence below 0.5% in questing *D. reticulatus*. This was also supported by a recent study failing to detect either *Borrelia* spp. or Anaplasmataceae in *D. reticulatus* from Berlin [62].

The overall frequency of pathogens in *I. hexagonus* was significantly higher than that in *I. ricinus* or *D. reticulatus.* This might be explained by the biology of *I. hexagonus*, which is a tick that lives in the mold of its host [63]. Female and male ticks live very closely together, and a homogenic distribution of infection rates is likely, since a lot of ticks share a small group of hosts living in the same mold.

Two or more pathogens were simultaneously present in as many as 20% of *I. ricinus* and 15% of *I. hexagonus.* Altough this does not necessarily mean that these ticks are truly co-infected, since some of the ticks might just have taken up the pathogens with their current blood meal, the very high number of ticks containing two pathogens suggests that simultaneous transmission of more than one pathogen by ticks is not a rare event. This is further corroborated by the fact that the vast majority of pathogens identified by sequencing to the species level, was obtained from ticks that are known vectors of these pathogens. Concurrent infections with more than one pathogen are of particular importance since they increase the risk of atypical forms of clinical disease [64]. A significantly increased frequency of *Rickettsia* spp. in *I. ricinus* infected with *A. phagocytophilum* than in *I. ricinus* not infected with *A. phagocytophilum* was found in this study. A possible explanation might be that an infection with *Rickettsia* spp. increases the susceptibility for *A. phagocytophilum* in ticks. Another explanation might be that both pathogens share the same reservoir hosts and that coinfection in reservoir hosts is also frequent [65]. *Ixodes ricinus* as a tick parasitizing on three different hosts during its life cycle can obtain more than one pathogen species from one or several hosts [66], and coinfections with *A. phagocytophilum* and *Rickettsia* spp. have been frequently described in ticks [65–70].

The SI provides a rough estimation of the feeding time from ticks on their hosts. Starting at a feeding time of 24 h, SIs are significantly different from one another when the time span between measurements is set to 24 h [34]. Particularly for ticks infected with pathogens that need at least 24 h feeding time for transmission to a new host, the SI is an important means to assess the infection risk of a host. Transmission times for pathogens detected are approximately 16 h for *Borrelia burgdorferi* s.l., 48 h for *Babesia* spp. and 24 h for Anaplasmataceae [71–73]. The shortest transmission times have been described for *Rickettsia ricketsii* with 10 h of feeding of *Dermacentor andersoni* in guinea pigs resulting in frequent transmission but even less than 2 h have been reported to be at least sometimes sufficient [74].

Comparison of the SI for ticks carrying a pathogen versus non-infected ticks revealed a statistically significant difference (p = 2.2 × 10^−7^) between both groups (Figure 2A and B); this was observed particularly for *D. reticulatus* harbouring *Rickettsia* spp. Ticks that have a long contact time with the host blood have a higher SI and a significantly higher probability harbouring an infectious agent. The fact that the SI was significantly higher in ticks infected with a pathogen strongly suggests that a considerable number of pathogen harboring ticks had obtained the pathogens from their current host. This information suggests that the prevalence of tick-borne pathogens in dogs in Berlin is high. The proposal is that a relatively high number of dogs harbour such pathogens at subclinical levels, which are not detectable by classical diagnostic methods, but are, nevertheless, sufficient for transmission to the vector. Alternatively, also pathogen reproduction within the tick during the feeding process might occur and lead to increased detection rates. Indirect serological methods are required to assess exposure of dogs to tick-transmitted pathogens, but such methods are currently only available for *Borrelia* spp. and *A. phagocytophilum* [75]. Due to their extremely high prevalence in ticks, *Rickettsia* spp. are presumably the most important tick-transmitted pathogens. However, concerning the *Rickettsia* species found in German ticks no data regarding the clinical importance in dogs are available yet.

## Conclusions

The results of this study are not only important in relation to dogs living in Berlin, but also for human health. In particular *I. ricinus* and *I. hexagonus* harbour a broad spectrum of pathogens and *I. ricinus* frequently attaches to humans as well [76]. The high pathogen frequency in ticks collected from dogs in Berlin/Brandenburg combined with the finding of emerging infectious disease agents, such as *Candidatus* Neoehrlichia mikurensis and *B. miyamotoi*, demonstrate the importance of continuous monitoring of tick populations for infectious pathogens. The development of inexpensive and effective diagnostic tools, such as PCR with high resolution melting curve analysis and identification of new gene markers that can be used for diagnostic purposes, should help to keep future studies cost- and time-efficient [23]. That the probability of ticks to harbour tick-borne pathogens increases with their feeding status (SI) calls for systematic screening of dogs for key tick-borne pathogens by both PCR and serology and for rigorous tick prophylaxis. The question as to whether *Rickettsia* spp. positive dogs are competent hosts for these zoonotic agents needs to be addressed experimentally.

## Additional files

Additional file 1: Table S1. Primers used for detection of pathogens. Additional file 2: Table S2. Raw data for all ticks used in the study. Additional file 3: Table S3. Ticks analysed for the presence of all pathogens. Additional file 4: Figure S1. Phylogenetic tree of *Borrelia* spp. sequences for *hbb* gene. The 105 bp sequence used for *B. miyamotoi* is from the present study and was deposited in the EBI database with accession number [HE993870]. Sequences were aligned using ClustalX2. The optimal nucleotide acid suptitution model was determined using jModeltest 0.1 before the maximum likelihood tree was calculated with Phyml 3.01 using the TPM3uf substitution model. Statistical support for individual branches is displayed with results of the Shimodaira-Hasegawa modification of the approximate likelihood ratio test before and of the baysian transformation of the approximate likelihood ratio test after the slash. The bar represents 0.02 substitutions per site. Accession numbers from Genbank® are given in brackets.
