# Supplementary material for: Pathogens in ticks collected from dogs in Berlin/Brandenburg, Germany
Source: Parasit Vectors. 2014 Dec 2;7:535. doi: 10.1186/s13071-014-0535-1 (PMC4262381; doi:10.1186/s13071-014-0535-1)
Supplement: Additional file 1: Table S1. — Primers used for detection of pathogens. [file 13071_2014_535_MOESM1_ESM.docx]

**Table S1**

Primers used for detection of pathogens

| **Pathogen (Amplicon size)** | **Sequence** | **Name** | **Reference** |
| --- | --- | --- | --- |
| ***Babesia* spp. (602-639 bp)** | GACACAGGGAGGTAGTGACAAG | RLB-F2 | Matjila et al., 2004 |
|  | AACCCAAAGACTTTGATTTCTCTC | B.mic1075lo |  |
| ***Rickettsia* spp.**  **(203 bp)** | GCTAAAGCTAAGGATAAAAATGAT  TCAATAAAATATTCATCTTTAAGAGC | Rmasglta 863up  Rmasglta 1065lo |  |
| ***Rickettsia* spp.**  **(676 bp)** | CCTATGGCTATTATGCTTGC  TCAATAAAATATTCATCTTTAAGAGC | CS409d  Rmasglta 1065lo | Roux et al., 2000 |
| ***Anaplasmataceae* (251 bp)** | GGGGATGATGTCAARTCAGCAY  CACCAGCTTCGAGTTAAGCCAAT | A/Efor  A/Erev | modifiedfromTabar et al., 2008 |
| ***Borrelia* spp. (153 bp)** | GTAAGGAAATTAGTTTATGTCTTT  TAAGCTCTTCAAAAAAAGCATCTA | Hbb640  HbbBw | Portnoi et al., 2006 |
| ***Borrelia* spp.**  **(600 bp)** | CTAACGCTGGCAGTGCGTCTTAAGC  AGCGTCAGTCTTGACCCAGAAGTTC  AGTCAAACGGGATGTAGCAATAC  GGTATTCTTTCTGATATCAACAG | 16S1A  16S1B  16S2A  16S2B | Richter et al., 2003 |
