# Supplementary material for: Pathogens in ticks collected from dogs in Berlin/Brandenburg, Germany
Source: Parasit Vectors. 2014 Dec 2;7:535. doi: 10.1186/s13071-014-0535-1 (PMC4262381; doi:10.1186/s13071-014-0535-1)
Supplement: Additional file 3: Table S3. — Ticks analysed for the presence of all pathogens. [file 13071_2014_535_MOESM3_ESM.docx]

**Table S3**

Ticks analysed for the presence of all pathogens

| Tick sex/stage | *I. ricinus* | *I. hexagonus* | *D. reticulatus*^a^ |
| --- | --- | --- | --- |
| Female | 169 | 100 | 105 |
| Male | 1 | 0 | 35 |
| Nymphs | 0 | 0 | 0 |
| Total | 170 | 100 | 140 |

^a^*D. reticulatus* were only subjected to PCR for Anaplasmatacea and *Rickettsia* spp.
