# Supplementary material for: Pathogens in ticks collected from dogs in Berlin/Brandenburg, Germany
Source: Parasit Vectors. 2014 Dec 2;7:535. doi: 10.1186/s13071-014-0535-1 (PMC4262381; doi:10.1186/s13071-014-0535-1)
Supplement: Additional file 4: Figure S1. — Phylogenetic tree of Borrelia spp. sequences for hbb gene. The 105 bp sequence used for B. miyamotoi is from the present study and was deposited in the EBI database with accession number [HE993870]. Sequences were aligned using ClustalX2. The optimal nucleotide acid suptitution model was determined using jModeltest 0.1 before the maximum likelihood tree was calculated with Phyml 3.01 using the TPM3uf substitution model. Statistical support for individual branches is displayed with results of the Shimodaira-Hasegawa modification of the approximate likelihood ratio test before and of the baysian transformation of the approximate likelihood ratio test after the slash. The bar represents 0.02 substitutions per site. Accession numbers from Genbank® are given in brackets. [file 13071_2014_535_MOESM4_ESM.pdf]

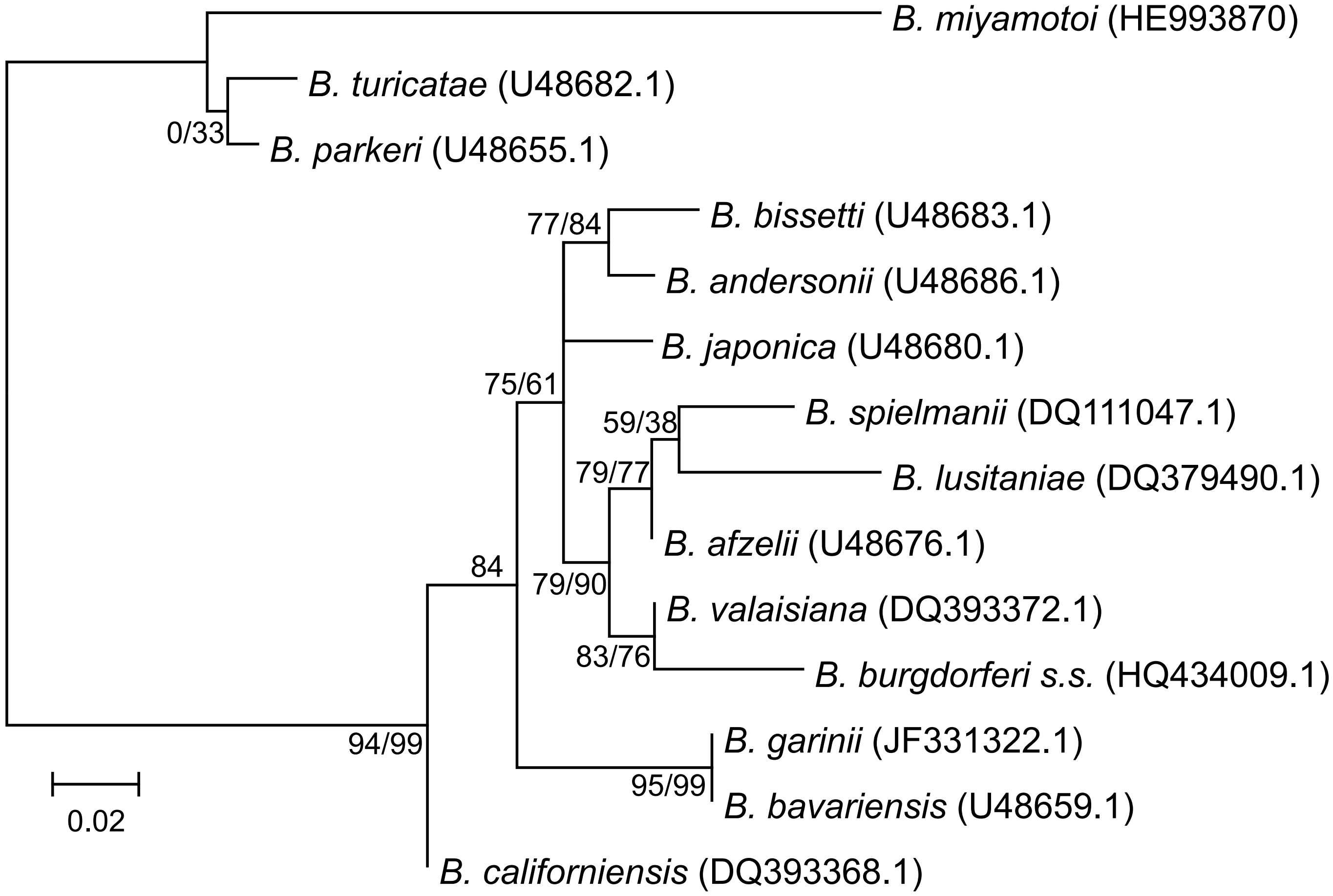

Figure S1 Phylogenetic tree of *Borrelia* spp. sequences for *hbb* gene. The 105 bp sequence used for *B. miyamotoi* is from the present study and was deposited in the EBI database with accession number [HE993870]. Sequences were aligned using ClustalX2. The optimal nucleotide acid suptitution model was determined using jModeltest 0.1 before the maximum likelihood tree was calculated with Phyml 3.01 using the TPM3uf substitution model. Statistical support for individual branches is displayed with results of the Shimodaira-Hasegawa modification of the approximate likelihood ratio test before and of the baysian transformation of the approximate likelihood ratio test after the slash. The bar represents 0.02 substitutions per site. Accession numbers from Genbank® are given in brackets.
